# Parliament and the Profession

**Published:** 1916-04-15

**Authors:** 


### PARLIAMENT AND THE PROFESSION.

### Infantile Mortality.

A statement bearing on a subject discussed in The Hospital last week (page 35) was made in the House of Commons by the President of the Local Government Board in answer to a question relating to infantile mortality. Mr. Long said that in spite of the general restrictions of local expenditure active steps are being taken by practically all the larger sanitary authorities to lessen infant mortality and to promote the welfare of infants generally. He mentioned that his department had distributed a grant of about £41,000 in aid of maternity and child welfare work during the financial year which has just ended; that a large number of local authorities had appointed health visitors, who advise mothers as to the care of their infants; and that in most of the large towns maternity and child welfare centres had been established.

### Rank Before Medical Qualification.

A question which called forth a reply which can have given little satisfaction to the questioner was asked by Mr. Shirley Benn. He inquired if young physicians and surgeons recognised as "modern practitioners" who join the Royal Army Medical Corps are made junior officers and have to take their orders from men who, "whether clever or stupid," have secured their promotion through length of service. Mr. Tennant's truly characteristic answer was, "If my hon. friend could provide an infallible criterion of distinguishing between clever and stupid people, and if he could secure its universal and willing acceptance by all the varied and numerous parties concerned, some beginning might be made to put in practice the Utopian system of his choice. In the meantime the answer to his question is that in the Army, as in most professions, the juniors take their orders from their seniors."

### Treatment of Tuberculous Soldiers.

The suggestion was made by Mr. R. McNeill that an arrangement should be made by which sailors and soldiers suffering from tuberculous disease may be kept undischarged for a period of not less than six months in hospitals specially reserved for them. The view of the War Office is that the results achieved by the existing system of treatment do not provide any justification for keeping soldiers undischarged for the long period suggested.

### The Supply of Artificial Limbs.

With regard to the provision of artificial limbs for disabled sailors and soldiers the idea seems to prevail in some quarters that the grants made by the Government for this purpose are inadequate to meet the needs of the case. Mr. W. Thorne, who voiced this opinion, also stated that many of the Poor-Law Guardians in different parts of the country had passed resolutions to the effect that the Government should take immediate steps to render all such expenses a national charge. According to the Financial Secretary to the War Office, however, artificial limbs are already supplied in kind at Government expense, and the recipients are fully maintained in hospital at public expense while they are being fitted.

### The Health of Munition Workers.

The recommendations of the Committee charged with the duty of investigating the health of the workers in war munitions, which were dealt with in The Hospital last week (p. 21), were the subject of several questions. Replying to Mr. Lewis Haslam, Mr. Lloyd George stated that the hours of work of women and young persons were limited by the regulations under the Factory Acts, but that the Ministry of Munitions was in regular communication with the Home Office as to the extent and circumstances in which orders of exemption should be granted with a view to giving effect to the recommendations of the Health of Munition Workers Committee. He added that no special instruction regulating the hours of male adult workers had been issued by the Ministry of Munitions, but that steps were being taken to reduce Sunday labour and to secure that adequate weekly rest periods were provided.

### Other Matters of Interest.

Lectures on Anti-typhoid Inoculation.—Mr. Chancellor raised the question whether Dr. Saleeby received any money from the Government for his lectures in favour of anti-typhoid inoculation. Mr. Tennant replied: "Dr. Saleeby, in giving the lectures referred to, was working voluntarily in aid of his fellow countrymen who are soldiers in His Majesty's Army."

Army Nurses' Allowances.—Replying to Sir Philip Magnus, who asked that the Order withdrawing from nurses on duty at the Front the allowances for lodging, fuel, and light might be repealed, the Financial Secretary to the War Office said he fully appreciated the services rendered by nurses, but he could hold out no hopes of any modification of the Order that had been issued.

Sickness at Oswestry Camp.—Replying to Sir Frederick Crawley, Mr. Tennant stated that the arrangements for dealing with cases of sickness at the Oswestry camp are the same as at other camps. The average strength at the camp during the last six months has been 9,269; during that period there have been 1,099 admissions to hospital, and twenty-seven deaths have taken place.

